# COVID‐19, ESG investing, and the resilience of more sustainable stocks: Evidence from European firms

**DOI:** 10.1002/bse.3163

**Published:** 2022-06-08

**Authors:** Giovanni Cardillo, Ennio Bendinelli, Giuseppe Torluccio

**Affiliations:** ^1^ Department of Management University of Bologna Bologna Italy; ^2^ Yunus Social Business Centre Forlì Italy; ^3^ Department of Management and Law University of Rome Tor Vergata Rome Italy

**Keywords:** corporate resilience, COVID‐19, ESG, ESG investing, stock market performance, stock markets, sustainability, sustainable finance

## Abstract

Following the COVID‐19 outbreak, orientation toward sustainability is a critical factor in ensuring firm survival and growth. Using a large sample of 1,204 firms in Europe during the year 2020, this study investigates how more sustainable firms fare during the pandemic compared with other firms in terms of risk–return trade‐off and stock market liquidity. We also highlight the drivers of the resilience of more sustainable firms to the pandemic. Particularly, we document that higher levels of cash holdings and liquid assets in the pre‐COVID period help these firms to perform and absorb the COVID‐19 externalities better than other firms. Our results are robust to a host of econometric models, including GMM estimations and several measures of stock market performance. These findings contribute to the theoretical and empirical debate on the role of the sustainability as a source of corporate resilience to unexpected shocks.

## 1 INTRODUCTION

The COVID‐19 pandemic has taken its toll on human lives and the real economy (Mirza et al., 2020; Reinhart, 2020), while financial markets worldwide grappled with significant disruptions (Zhang et al., 2020). On February 24, 2020—the first trading day after lockdown measures in Northern Italy—many stock exchanges have experienced their hardest times since the Great Financial Crisis of 2008–2009 (Ramelli & Wagner, 2020). In these circumstances, the pandemic has also highlighted more than ever the importance of the firm sustainability performance as one of the main drivers of the firm resilience to unexpected shocks (Albuquerque et al., 2020; Barro et al., 2020; Koçak et al., 2021; Ramelli & Wagner, 2020) by attracting the interest of consumers, investors,^1^ asset managers, and policymakers as well as scholars (Amundi Asset Management, 2020; Cunha et al., 2021; Global Sustainable Investment Alliance, 2020). On the one hand, national and central governments spurred the sustainable transition by including environmental and social priorities into crisis management at the national level (JP Morgan, 2020; Wells Fargo, 2020).^2^ On the other hand, asset managers played a central role prioritizing the integration of ESG factors into investment solutions in line with the fast‐growing interest and changes in preferences of international investors (Friede, 2019; JP Morgan, 2020).

In this context, several studies have investigated the firm stock‐market performance considering their orientation toward many aspects of sustainability by finding inconclusive results and discrepancies. Bae et al. (2021) find no evidence that corporate social responsibility affect stock market returns during the pandemic. Garel and Petit‐Romec (2021) find that more environmentally friendly firms are likely to experience better stock returns, while Albuquerque et al. (2020) find that non‐financial firms with higher environmental and social impact scores show higher returns and lower return volatilities. The reason for such a lack of consensus might be driven by factors related to the sample period under investigation (for instance, first quarter of 2020 rather than specific time windows of the pandemic), the geographical area under consideration (for example, US firms rather than EU firms), different econometric strategies as well as the type of economic agents considered by prior studies (i.e., financial firms and non‐financial firms). Similar to our research, Ding et al. (2021) find that more sustainable firms experienced a milder drop in their weekly stock returns than other firms. However, in terms of econometric setup, they rely on weekly data and cover a period from the beginning of January through the end of May 2020. Notably, none of the prior studies examines the link between the firm sustainability performance and stock market performance during the whole year of 2020 (from January 2020 to December 2020) by using different constituent pillars of the ESG ratings (Koçak et al., 2021), different statistical strategies (Cunha et al., 2020), different economic agents (financial and non‐financial firms), and different geographic areas. For instance, most of them are either one‐country setting studies (Albuquerque et al., 2020; Aslam et al., 2021; Atif & Ali, 2021; Zhou et al., 2022) or sector‐specific (Bhandari et al., 2022). Moreover, although Ding et al. (2021) consider a sample of 6700 firms across 61 economies, our sample is more homogenous in terms of the institutional framework since we focus on Western Europe (1,204 firms). Furthermore, we are also the first to study the implications of the COVID‐19 spread in relation to the firm's stock liquidity. Hence, we seek to respond to the following research questions: how do highly rated ESG score firms perform during the COVID‐19 shock? Why do they perform better than other firms during a pandemic shock?

Hence, the contribution of this paper to the extant literature is threefold. First, we examine the effects of COVID‐19 on the more sustainable firms' stock market performance. While there is already literature on the effects of each pillar of the ESG rating on the financial performance (Barnett & Salomon, 2012; Freeman & Reed, 1983; Margolis et al., 2009; Porter & Kramer, 2006; Schnippering, 2020; Semenova & Hassel, 2008; Ullmann, 1985; Waddock & Graves, 1997), there is no evidence on the performance of more sustainable firms—financial and non‐financial firms—during the whole and most feverish period of the pandemic (2020) using daily data. Since our results suggest firms with higher ESG scores perform better than other firms during the pandemic, this evidence is also coherent with the view that sustainability performance might serve as an insurance‐like instrument against adverse economic events (Godfrey, 2005; Godfrey et al., 2009).

Second, we also speak to the literature on the firm resilience to unexpected economic shocks (Albuquerque et al., 2020). Notably, we investigate whether the better stock market performance of highly rated ESG score firms is related to either the firm's ex‐ante liquidity conditions or firm profitability in the pre‐COVID period. Our estimates suggest that the more sustainable firm resilience is built on the ex‐ante liquidity conditions, which matter not only for firm financial obligations, but also to absorb the COVID‐19 externalities.

Third, this research is one of the first attempts to provide novel evidence on the relationship between firm sustainability and stock market performance in European countries. Past studies focus mainly on the US market (among others, Albuquerque et al., 2020; Ramelli & Wagner, 2020). Yet, this facet provides us an important space for contribution for two reasons. On the one hand, as well as institutional, macroeconomic, and market differences between United States and Europe (Allen et al., 2004), Europe is committed to the UN's 2030 Agenda, aimed at the climate‐neutrality by 2050, and is one of the most active players toward the Sustainable Development Goals (SDGs).^3^ On the other hand, the then US President—Donald Trump—encouraged local governors to terminate local stay‐at‐home restrictions, while European countries showed more proactive management of the pandemic mobilizing promptly financial resources to households and corporates to contain the economic effects.^4^ ^,^ ^5^

We find two important results. First, we find that more sustainable firms have a better stock market performance than other firms during the pandemic since they show higher market returns, lower volatilities, and higher stock market liquidity. We refer to the latter variable as the ease with which a stock may be converted into cash without incurring losses in the market value, often under‐investigated in previous studies even if investors show a liquidity search behavior in the stock‐picking process (Amihud & Mendelson, 1989). Moreover, this result is stronger when we account for the type of the economic agent considered (for instance, financial firms, non‐financial firms, and the exclusion of oil‐related firms' observations), firm heterogeneity (firm‐fixed effects), the daily Fama French, and daily macroeconomic factors. We also allow for the dynamic endogeneity in our estimates.

Second, we highlight the mechanism through which highly rated ESG score firms perform better than others during the pandemic crisis. Particularly, our findings suggest that more sustainable firms retaining higher levels of cash holdings and liquid assets in their balance sheets in the pre‐COVID period are more likely to absorb better than others the pandemic shock. This result is in line with Ding et al. (2021). Interestingly, this finding is stronger for financial firms rather than non‐financial firms.

This paper is organized as follows. Section 2 describes the related literature and illustrates the hypotheses development. Section 3 introduces the methodology and the data used for our main tests. Section 4 discusses the results, robustness tests, and extensions. Section 5 reports our concluding remarks.

## 2 RELATED LITERATURE AND TESTABLE HYPOTHESES

### 2.1 Literature review

This paper is related to two different strands of literature. The closer strand is the bourgeoning body of research on the impact of the COVID‐19 pandemic on financial markets (Ramelli & Wagner, 2020; Zhang et al., 2020). The COVID‐19 has had significant implications for global financial markets. Most studies highlight that during the COVID‐19 pandemic shock, because of the market crash, stock prices experience negative returns and higher volatilities (Ashraf, 2020; Erdem, 2020; Mazur et al., 2020; Zhang et al., 2020). However, Ramelli and Wagner (2020) and Albuquerque et al. (2020) find that stock price movements follow different paths during each phase of the COVID‐19 outbreak. Furthermore, previous studies underline that stock sensitivity to this pandemic may vary because of several factors, such as sector elasticity to the COVID‐19 (Mazur et al., 2020; Ramelli & Wagner, 2020), trade interconnections either with China or with other countries most affected by the pandemic (Ding et al., 2021; Ramelli & Wagner, 2020), and the country degree of freedom (Erdem, 2020).

The second strand of the literature that this paper is referred to is the broader body of studies related to the relationship between firm sustainability and firm performance (Minutolo et al., 2019; Qureshi et al., 2020; Xie et al., 2019). This literature postulates fragmented findings invoking different theoretical frameworks, such as the stakeholder theory,^6^ the resource‐based view (Bhandari et al., 2022),^7^ and the legitimacy theory (Friede et al., 2015; Whelan et al., 2021).^8^ For instance, Vishwanathan et al. (2020) suggest that sustainability‐oriented initiatives aimed at supporting either the stakeholder reciprocation or the firm innovation capacity lead to higher financial performance, while Dorfleitner et al. (2018) suggest that the effect of sustainability‐oriented initiatives is more pronounced in the long‐term run rather than in the short‐term run.

Yet, some studies also support the view that firm sustainability orientation is not necessarily associated with a better corporate financial performance (Friede et al., 2015). Revelli and Viviani (2015) and Humphrey et al. (2012) find no evidence that firms with higher ESG ratings have a better risk‐adjusted performance. Di Giuli and Kostovetsky (2014) and Lys et al. (2015) find that increases in the CSR scores and expenditures are associated with lower corporate financial performance. Plausible explanations for this evidence are related to the managerial entrenchment (Surroca & Tribó, 2008) and potential agency problems in the firm philanthropy activities (Masulis & Reza, 2015).

### 2.2 Hypotheses development: COVID‐19 cases and deaths, ESG ratings, and stock market returns

The onset of the COVID‐19 severely affects financial markets and firm stock performance. Stock market performance sensitivity to economic shocks may change in relation to the firm sustainability performance (Albuquerque et al., 2020). This evidence is well‐supported in the literature considering both financial and non‐financial firms and different economic shocks occurred in the financial system. For instance, Lins et al. (2017) demonstrate that firms with higher social capital, proxied by the firm's corporate social responsibility, show higher stock returns than firms with lower social capital during the last 2007–08 financial crisis. This result is also confirmed by Cornett et al. (2016) and Cheema‐Fox et al. (2021). Albuquerque et al. (2020) show that the better market performance is because firms with higher ESG scores are more resilient to the shocks because of customer and investor loyalty (Albuquerque et al., 2019). In turn, this view is also coherent with Whelan et al. (2021), suggesting that these stocks better protect against the downside risk for the sake of the portfolio construction. Additionally, in the spirit of Ferrell et al. (2016), given that higher ESG scores reflect both business strategies concerning ESG factors and firm quality of the top‐management, it is also plausible that well‐governed firms might have a better performance during the pandemic and investors might prefer having stocks of these firms in their portfolios. This would shift investors' preferences toward stocks of firms with higher ESG scores and enhance their stock market performance. This is coherent with prior studies on *flight‐to‐quality*,^9^ namely, the possibility that when the uncertainty over financial markets rises, investors shift their portfolios from riskier to less risky assets during economic downturns.

In contrast with this set of studies, there is also some evidence according to which the relationship between firm sustainability and stock market performance is not necessarily positive. For instance, Demers et al. (2021) find that firms with higher ESG ratings have a lower market performance than other firms during bad states of the economy, while Bae et al. (2021) find no evidence that corporate social responsibility affects stock market returns during the pandemic.

Based on these conflicting arguments, we posit the following alternative hypotheses:

Firms with higher ESG scores have higher stock market performance (higher returns and lower volatility) than firms with low ESG scores when COVID‐19 cases increase.

Firms with higher ESG scores have lower stock market performance than firms with low ESG scores when COVID‐19 cases increase.

Firms with higher ESG scores have a stock market performance (higher returns and lower volatility) in line with firms with low ESG scores when COVID‐19 cases increase.

## 3 METHODOLOGY AND DATA

This section provides a detailed description of the methodology and the data set, and then it describes the summary statistics.

### 3.1 Methodology

In the spirit of Erdem (2020), we first correlate the firm's daily stock performance (*Perf*) with the corresponding COVID‐19 confirmed cases (*Cases*) by using the following panel data model regression:

(1) $${\text{Perf}}_{i,t}=b_{0}+b_{1}\;\text{Highly}-\text{rated}\;\mathrm{ESG}\;{\text{firm}}_{2019}+b_{2}\;{\text{Cases}}_{c,t}+b_{3}\;\text{Highly}-\text{rated}\;\mathrm{ESG}\;{{\text{firm}}_{2019}}^{*}\;{\text{Cases}}_{c,t}+b_{4}\;\mathrm{Day}\;{\mathrm{FE}}_{t}+e_{i,t}$$

where *i* indexes for the stock returns of the firm *i* and *t* indexes for the trading days.

Our empirical framework uses several variables of the market performance measured over the period starting from January 1, 2020 to December 31, 2020: daily log‐returns (*Raw returns*), the market‐adjusted returns (*Market‐adjusted returns*), the excess returns (*Excess returns*), and the 5‐day moving volatility (*Volatility*). The daily log returns for each stock *i* on day *t* are calculated as follows:

(2) $$\mathrm{Raw}\;{\text{Returns}}_{i,t}=\ln\;\left({\text{Price}}_{i,t}/{\text{Price}}_{i,t-1}\right)$$

where *Price*_i,t_ stands for the closing price of stock *i* in day *t*.

Second, we rely on *Market‐adjusted returns* defined as the difference between the log‐returns of the firm stock prices and the log‐returns of the reference national market index. Third, we employ *Excess returns*, calculated as the difference between the daily return earned by a given stock minus the risk‐free rate. In line with Fama and French (1992, 1993), we use the short‐term government treasury bills (*Risk Free Rate*) as a risk‐free asset. More formally, we estimate excess returns according to the following formula:

(3) $${\text{Excess Returns}}_{i,t}=\mathrm{Raw}\;{\text{Returns}}_{i,t}–\text{Risk}\;{\text{Free Rate}}_{t}$$

The advantage of this measure lies in the fact that it compares the stock market performance with a risk‐free alternative. Thus, investors would prefer investments with a positive excess return since the riskier investment strategy provides a higher reward than they could obtain if investing in a risk‐free asset.

Then, we calculate the 5‐day moving return volatility (Erdem, 2020) as

(4) $$\text{Volatility}=\sqrt{\frac{\sum_{t=1}^{5}{\left({\text{Returns}}_{i,t}-{\bar{\text{Returns}}}_{i}\right)}^{2}}{4}}$$

where $\bar{\text{Returns}}$ stands for the mean return of stock *i*.

Hereafter, we turn our attention to three key variables of our estimation procedure (*Highly rated ESG firm*_*2019*_, *Cases*, and *Deaths*). First, in line with Albuquerque et al. (2020), *Highly rated ESG firm*_*2019*_ is a time‐invariant dummy variable that takes the value of one if the firm has an ESG rating higher than the median firm of the population of EU‐firms in 2019 and zero otherwise. This allows us to exclude any simultaneity concerns and spurious correlation with the outcome variables and understand how highly rated ESG firms in 2019 step into the pandemic crisis. Then, we include *Cases*, which is estimated as the log‐growth of confirmed coronavirus cases in the country *c*, where the firm has its headquarters in the day *t*. We use the following formula:

(5) $${\text{Cases}}_{i,t}=\ln\;\left({\text{Confirmed Cases}}_{c,t}/{\text{Confirmed Cases}}_{c,t-1}\right)$$

In some specifications, we also replace the variable *Cases* with *Deaths*, which is similarly defined as the daily log‐growth of deaths in the country *c* in the day *t*:

(6) $${\text{Deaths}}_{i,t}=\ln\;\left(\text{COVID}-19\;{\text{Deaths}}_{c,t}/\text{COVID}-19\;{\text{Deaths}}_{c,t-1}\right)$$

### 3.2 Data

This study relies on a data set of all non‐financial and financial companies having an available ESG rating in Refinitiv and listed in the EU‐14 European countries (Austria, Belgium, Denmark, Finland, France, Germany, Greece, Ireland, Italy, Luxembourg, Netherlands, Portugal, Spain, and Sweden) and the United Kingdom(Cardillo et al., 2021). We choose a sample of EU‐14 countries and the United Kingdom, because they share both a uniform financial regulatory framework and a similar government monitoring on listed firms (Onali et al., 2016; Pattitoni et al., 2014). The final sample includes 1,204 European firms (financial firms = 375; non‐financial firms = 829) and 227,923 firm‐day observations, as suggested by Table 1 and Table 2.

**TABLE 1:** Sample composition by country

Country Name	Number of observations	%	Number of firms	%	Number of non‐financial firms	%	Number of financial firms	%
Austria	5,780	2.54	31	2.57	20	2.41	11	2.93
Belgium	8,088	3.55	44	3.65	24	2.9	20	5.33
Denmark	8,431	3.7	44	3.65	34	4.1	10	2.67
Finland	8,561	3.76	45	3.74	34	4.1	11	2.93
France	26,439	11.6	133	11.05	100	12.06	33	8.8
Germany	27,270	11.96	152	12.62	96	11.58	56	14.93
Greece	3539	1.55	21	1.74	13	1.57	8	2.13
Ireland	4,368	1.92	21	1.74	16	1.93	5	1.33
Italy	18,806	8.25	98	8.14	64	7.72	34	9.07
Luxembourg	2,697	1.18	16	1.33	7	0.84	9	2.4
Netherlands	9,352	4.1	49	4.07	33	3.98	16	4.27
Portugal	2,633	1.16	14	1.16	11	1.33	3	0.8
Spain	11,608	5.09	62	5.15	45	5.43	17	4.53
Sweden	28,853	12.66	150	12.46	106	12.79	44	11.73
United Kingdom	61,498	26.98	324	26.91	226	27.26	98	26.13
Total	227,923	100	1,204	100	829	100	375	100

*Note*: This table shows the sample composition by country.

**TABLE 2:** Sample composition by economic sector

Economic sector	Number of firms	Percentage
Academic and educational services	2	0.17
Basic materials	94	7.81
Consumer cyclicals	200	16.61
Consumer non‐cyclicals	94	7.81
Energy	40	3.32
Financials	167	13.87
Healthcare	88	7.31
Industrials	266	22.09
Real estate	81	6.73
Technology	127	10.55
Utilities	45	3.74
Total	1,204	100

*Note*: This table shows the sample composition by economic sector.

We collect data from multiple data sources. First, we collect information on stock prices from Datastream. Since we rely on Fama French factors in some robustness tests, we download information on the firm's stock prices in US dollars rather than euros. Second, we borrow information on the firm's ESG scores from Refinitiv database (Ding et al., 2021). In comparison with other ESG rating providers (Widyawati, 2020), Refinitiv, as well as being one of the largest providers of ESG performance for firms, shows a better coverage of the European market than other providers (for instance, KLD). Furthermore, it also presents an economic estimate about the firm's ability to create shareholder value and contribution to sustainable growth (Berg et al., 2020; Dorfleitner et al., 2015). Finally, Refinitiv considers a most extensive range of indicators and dimensions to construct each constituent component of the aggregate indicator related to firm sustainability performance (Berg et al., 2020; Dorfleitner et al., 2018).^10^ Finally, we also collect information on COVID‐19 cases and deaths from the Oxford University COVID‐19 Government Response Tracker.

Table 3 summarizes the descriptive statistics. In particular, we report the mean, the standard deviation, and the range of each variable used in our estimation procedure. Overall, the descriptive statistics show a high heterogeneity and variability.

**TABLE 3:** Summary statistics

Variables	Obs.	Average	Std. dev.	Min.	Max.
Dependent variable(s)					
*Raw returns*	227,923	0.0008	0.0375	−1.4917	1.5163
*Market‐adjusted returns*	227,923	0.0003	0.0332	−1.4867	1.5031
*Excess returns*	227,923	0.0001	0.0380	−1.4917	1.5163
*Volatility*	227,864	0.0262	0.0190	0.0036	0.3934
Variables of interest					
*Highly rated ESG firm*_*2019*_	227,923	0.4305	0.4951	0.0000	1.0000
*Cases*	227,923	0.0412	0.1047	−0.2417	2.4380
*Deaths*	227,923	0.0487	0.1517	−0.2377	1.9459
Other control variables					
*Rm‐Rf*	227,923	0.1273	1.8396	−12.0000	8.5400
*SMB*	227,923	0.0394	0.6315	−3.3300	1.8400
*HML*	227,923	−0.0827	0.9532	−3.0400	4.3800
*RMW*	227,923	0.0226	0.3164	−0.7900	0.9300
*CMA*	227,923	−0.0757	0.4219	−1.2000	1.3100
*WML*	227,923	0.0051	1.5373	−10.8700	3.6600
*Stringency Index*	227,923	0.6306	0.1420	0.0556	0.9074
*Health Index*	227,923	0.5832	0.1045	0.0952	0.8363
*Economic Index*	227,923	0.7397	0.2597	0.0000	1.0000

*Note*: This table reports the descriptive statistics for all the variables used in the main regression analyses. The sample period is from January 1, 2020 to December 31, 2020. For each variable, we show the following statistics: number of observations (*Obs*.), mean (*Average*), standard deviation (*Std. dev*.), minimum value (*Min*.), and maximum value (*Max*.). The dependent variables are daily log‐returns (*Raw returns*), market‐adjusted returns (*Market‐adjusted returns*), excess returns (*Excess returns*), and the 5‐day rolling return volatility (*Volatility*), respectively. *Highly rated ESG firm*_*2019*_ is a dummy variable taking the value of one if the firm has a ESG rating higher than the median ESG score of the population of listed firms in the year before the COVID‐19 pandemic (2019). *Cases* variable is the daily log‐growth of confirmed coronavirus cases in the country *c* in the day *t*. *Deaths* is the daily log‐growth of deaths in the country *c* in the day *t*. *Rm‐rf*, *SMB*, *HML*, *RMW*, and *CMA* are the Fama French factors. *WML* is a momentum factor. *Stringency Index*, *Health Index*, *Economic Index* are the Stringency Index, the Containment and Health Index, and Economic Support Index from Oxford Covid‐19 Government Response Tracker. All variables are winsorized at the 1st and 99th percentiles except *Raw returns*, *Cases*, and *Deaths* because they are log‐variables.

## 4 RESULTS

### 4.1 Main results: confirmed cases, deaths, and highly rated ESG firms stock performance

In this subsection, we investigate the results of the panel data regressions based on a random‐effects model that correlates log‐daily returns, market‐adjusted returns, excess returns, and the five‐day moving volatility with the log‐growth of COVID‐19 confirmed cases (*Cases*) and deaths (*Deaths*). We report the results in Table 4.

**TABLE 4:** COVID‐19 and stock market performance of highly rated ESG firms

Variables	Raw returns	Market‐adjusted returns	Excess returns	Volatility	Raw returns	Market‐adjusted returns	Excess returns	Volatility
	(1)	(2)	(3)	(4)	(5)	(6)	(7)	(8)
*Highly rated ESG firm*_*2019*_	−0.0003*** (−2.7515)	−0.0002** (−2.2048)	−0.0003*** (−2.7515)	−0.0035*** (−3.6920)	−0.0004*** (−3.5592)	−0.0004*** (−3.2275)	−0.0004*** (−3.5592)	−0.0035*** (−3.7051)
*Cases*	−0.0042** (−2.4396)	0.0016 (0.9381)	−0.0042** (−2.4396)	0.0004 (1.4393)				
*Deaths*					−0.0086*** (−4.0057)	−0.0110*** (−5.1563)	−0.0086*** (−4.0057)	0.0000 (0.2155)
*Highly rated ESG firm*_*2019*_ * *Cases*	0.0032 (1.5908)	0.0024 (1.2039)	0.0032 (1.5908)	−0.0009* (−1.9182)				
*Highly rated ESG firm*_*2019*_ * *Deaths*					0.0046** (2.4844)	0.0043** (2.3554)	0.0046** (2.4844)	−0.0006 (−1.5442)
Intercept	Yes	Yes	Yes	Yes	Yes	Yes	Yes	Yes
Observations	227,923	227,923	227,923	227,864	227,923	227,923	227,923	227,864
*R*‐squared	0.267	0.070	0.284	0.100	0.267	0.071	0.284	0.100
Number of firms	1,204	1,204	1,204	1,204	1,204	1,204	1,204	1204
Sector FEs	Yes	Yes	Yes	Yes	Yes	Yes	Yes	Yes
Day FEs	Yes	Yes	Yes	Yes	Yes	Yes	Yes	Yes

*Note*: This table shows the panel regression results for stock returns and volatility for the period January 2020 to December 2020. The dependent variables are daily log‐returns (*Raw returns*), market‐adjusted returns (*Market‐adjusted returns*), excess returns (*Excess returns*), and the 5‐day rolling return volatility (*Volatility*) respectively. *Highly rated ESG firm*_*2019*_ *is* a dummy variable taking the value of one if the firm has a ESG rating higher than the median ESG score of the population of listed firms in the year before the COVID‐19 pandemic (2019). *Cases* variable is the daily log‐growth of confirmed coronavirus cases in the country *c* in the day *t*. *Deaths* is the daily log‐growth of deaths in the country *c* in the day *t*. Regressions include day and sector fixed effects because ESG ratings differ across sectors. Robust t‐statistics are reported in parentheses.

* *p* < 0.1.

** *p* < 0.05.

*** *p* < 0.01.

In line with Erdem (2020) and Ding et al. (2021), our results show that when the log‐growth of the number of COVID‐19 confirmed cases increases in the economy, all the firms in our sample show a lower stock market performance (*Raw returns*). This result is also robust when we consider the variable of *Excess returns* ^11^ and when we replace *Cases* with *Deaths*.^12^

Second, we move onto the coefficients of our interest, namely *Highly rated ESG firm*_*2019*_ * *Cases* and *Highly rated ESG firm*_*2019*_ * *Deaths*. On the one hand, we find that the coefficient on *Highly rated ESG firm*_*2019*_ * *Cases* is not statistically significant when we consider *Raw returns*, *Market‐adjusted returns*, and *Excess returns*, suggesting that highly rated ESG score firms are not sensitive to the spread of the pandemic over the whole sample period. However, we find that the coefficient on *Highly rated ESG firm*_*2019*_ * *Cases* for *Volatility* is weakly significant at 10% level suggesting that these stocks show lower volatilities than firms with lower ESG scores.

Interestingly, when we replace *Cases* with *Deaths*, we find opposite evidence. On the one hand, we find that highly rated ESG score firms perform better than low‐rated ESG score firms across all specifications (*Raw returns*, *Market‐adjusted returns*, *Excess returns*) when the COVID‐19 deaths increase in the economy. Considering the coefficient on *Highly rated ESG firm*_*2019*_ * *Deaths* in Column 5, it suggests that an increase by one standard deviation in the log‐growth of COVID‐19 deaths would increase *Raw Returns* by 0.07 percentage points at daily basis for firms with higher ESG scores. Given that for each stock, on average, we have 260 trading days, we obtain that the annual effect is 112 percentage points (alternatively, 1.12% = $\sqrt{260}*0.07*100$). This finding is consistent with Heyden and Heyden (2021). On the other hand, the coefficient on *Highly rated ESG firm*_*2019*_ * *Deaths* for *Volatility* is not statistically significant.

Overall, our evidence suggests that highly rated ESG score firms perform better than low‐rated ESG score firms. Since the results are not entirely consistent across all specifications, this brings us to explore further our estimates by allowing for the firm‐heterogeneity, daily macroeconomic factors, Fama French factors, momentum, and dynamic endogeneity.

### 4.2 Robustness checks and extensions

#### 4.2.1 Firm heterogeneity

When analyzing the effects of COVID‐19 cases and deaths on the stock market performance of highly rated ESG score firms, endogeneity concerns may arise because of the omitted and unobserved firm characteristics. Dealing with daily data entails that we do not have any information on firm characteristics and fundamentals affecting the firm's stock market performance. Hence, omitted variables might lead to spurious correlations between firm sustainability performance in 2019 and the firm's stock market performance during the COVID‐19 pandemic. Indeed, one may argue that some firms might be more progressive than others, or simply, the existence of other time‐constant firm characteristics—for instance, the corporate culture.^13^ This aspect may drive the higher stock market performance of more sustainable firms. Thus, we allow for firm‐fixed effects (Adams & Ferreira, 2009).

In Table 5, we repeat our main regression analysis including firm‐fixed effects. The results reiterate previous results reported in Table 4 for the interaction term coefficient on *Highly rated ESG firm*_*2019*_ * *Deaths* in relation to the stock market variables. In contrast, when considering the coefficient on *Highly rated ESG firm*_*2019*_ * *Cases* is still positive in Column 1 and Column 3, it becomes statistically significant at 10% level. The coefficient in Column 1 suggests that an increase by one standard deviation in the log‐growth of COVID‐19 cases would increase daily *Raw Returns* by 0.04 percentage points for firms with higher ESG scores. It entails that firms with higher ESG scores annually gain almost 57 percentage points, while if we consider the result in Column 4, we obtain that highly rated ESG score firms have lower volatility lower than minus 15 percentage points (0.15%).

**TABLE 5:** Robustness tests: COVID‐19, stock market performance of highly rated ESG firms, and firm‐fixed effects

Variables	Raw returns	Market‐adjusted returns	Excess returns	Volatility	Raw returns	Market‐adjusted returns	Excess returns	Volatility
	(1)	(2)	(3)	(4)	(5)	(6)	(7)	(8)
*Cases*	−0.0040** (−2.3150)	0.0018 (1.0462)	−0.0040** (−2.3150)	0.0004 (1.4380)				
*Deaths*					−0.0087*** (−4.0224)	−0.0111*** (−5.1424)	−0.0087*** (−4.0224)	0.0000 (0.2140)
*Highly rated ESG firm*_*2019*_ * *Cases*	0.0034* (1.7387)	0.0025 (1.3021)	0.0034* (1.7387)	−0.0009* (−1.9179)				
*Highly rated ESG firm*_*2019*_ * *Deaths*					0.0048** (2.5625)	0.0045** (2.4033)	0.0048** (2.5625)	−0.0006 (−1.5439)
Intercept	Yes	Yes	Yes	Yes	Yes	Yes	Yes	Yes
Observations	227,923	227,923	227,923	227,864	227,923	227,923	227,923	227,864
*R*‐squared	0.267	0.070	0.283	0.191	0.267	0.071	0.283	0.191
Number of firms	1,204	1,204	1,204	1,204	1,204	1,204	1,204	1,204
Firm FEs	Yes	Yes	Yes	Yes	Yes	Yes	Yes	Yes
Day FEs	Yes	Yes	Yes	Yes	Yes	Yes	Yes	Yes

*Note*: This table shows the panel regression results for stock returns and volatility for the period January 2020 to December 2020. The dependent variables are daily log‐returns (*Raw returns*), market‐adjusted returns (*Market‐adjusted returns*), excess returns (*Excess returns*), and the 5‐day rolling return volatility (*Volatility*) respectively. *Highly rated ESG firm*_*2019*_ is a dummy variable taking the value of one if the firm has a ESG rating higher than the median ESG score of the population of listed firms in the year before the COVID‐19 pandemic (2019). *Cases* variable is the daily log‐growth of confirmed coronavirus cases in the country *c* in the day *t*. *Deaths* is the daily log‐growth of deaths in the country *c* in the day *t*. Regressions include firm‐ and day‐fixed effects. Robust *t* statistics are reported in parentheses.

* *p* < 0.1.

** *p* < 0.05.

*** *p* < 0.01.

#### 4.2.2 Sample splits: Financial firms versus non‐financial firms

Although we include sector‐fixed effects in our main analysis (Table 4) to account for systematic differences in risk and stock market performance across sector‐types, financial firms substantially differ from non‐financial firms. For instance, financial firms are more likely to threaten the financial stability and determine systemic issues than other firms in case of their bankruptcy (Bell & Keller, 2009; Geneva Association, 2010; International Association of Insurance Supervisors, 2012; Mühlnickel & Weiβ, 2015; Stern & Feldman, 2004) and create feedback loop effect in the public budgets (Acharya et al., 2014). Thus, we split our sample into financial and non‐financial institutions and re‐run our regressions in Table 4. Table 6 reports the results.

**TABLE 6:** Robustness tests: Sample splits for financial and non‐financial firms

Variables	Raw returns	Market adjusted returns	Excess returns	Volatility	Raw returns	Market adjusted returns	Excess returns	Volatility
	(1)	(2)	(3)	(4)	(5)	(6)	(7)	(8)
**Panel A: Non‐financial firms**								
*Highly rated ESG firm*_*2019*_	−0.0002 (−1.3806)	−0.0001 (−1.0946)	−0.0002 (−1.3806)	−0.0042*** (−3.1271)	−0.0003* (−1.9409)	−0.0003* (−1.7657)	−0.0003* (−1.9409)	−0.0042*** (−3.1306)
*Cases*	−0.0052** (−2.4136)	0.0006 (0.3034)	−0.0052** (−2.4136)	0.0002 (0.4818)				
*Deaths*					−0.0126*** (−4.6983)	−0.0150*** (−5.6227)	−0.0126*** (−4.6983)	−0.0002 (−0.5307)
*Highly rated ESG firm*_*2019*_ * *Cases*	0.0054** (2.1295)	0.0044* (1.7744)	0.0054** (2.1295)	−0.0004 (−0.7086)				
*Highly rated ESG firm*_*2019*_ * *Deaths*					0.0062*** (2.6492)	0.0056** (2.3922)	0.0062*** (2.6492)	−0.0004 (−0.7051)
Intercept	Yes	Yes	Yes	Yes	Yes	Yes	Yes	Yes
Observations	155,125	155,125	155,125	155,078	155,125	155,125	155,125	155,078
*R*‐squared	0.257	0.068	0.274	0.098	0.258	0.069	0.275	0.098
Number of firms	829	829	829	829	829	829	829	829
Sector FEs	Yes	Yes	Yes	Yes	Yes	Yes	Yes	Yes
Day FEs	Yes	Yes	Yes	Yes	Yes	Yes	Yes	Yes
**Panel B: Non‐financial firms with the exclusion of oil‐ and energy‐related firms**								
*Highly rated ESG firm*_*2019*_	−0.0002 (−1.2326)	−0.0001 (−0.9798)	−0.0002 (−1.2326)	−0.0036*** (−2.6191)	−0.0003* (−1.7949)	−0.0002* (−1.6712)	−0.0003* (−1.7949)	−0.0036*** (−2.6214)
*Cases*	−0.0049** (−2.2100)	0.0008 (0.3842)	−0.0049** (−2.2100)	0.0003 (0.9243)				
*Deaths*					−0.0117*** (−4.2875)	−0.0144*** (−5.3024)	−0.0117*** (−4.2875)	−0.0000 (−0.0248)
*Highly rated ESG firm*_*2019*_ * *Cases*	0.0040 (1.5899)	0.0031 (1.2424)	0.0040 (1.5899)	−0.0007 (−1.0733)				
*Highly rated ESG firm*_*2019*_ * *Deaths*					0.0051** (2.1610)	0.0046* (1.9487)	0.0051** (2.1610)	−0.0006 (−1.0831)
Intercept	Yes	Yes	Yes	Yes	Yes	Yes	Yes	Yes
Observations	148,922	148,922	148,922	148,875	148,922	148,922	148,922	148,875
*R*‐squared	0.267	0.073	0.285	0.096	0.268	0.074	0.285	0.096
Number of firms	790	790	790	790	790	790	790	790
Sector FEs	Yes	Yes	Yes	Yes	Yes	Yes	Yes	Yes
Day FEs	Yes	Yes	Yes	Yes	Yes	Yes	Yes	Yes
**Panel C: Financial firms**								
*Highly rated ESG firm*_*2019*_	−0.0005*** (−3.1246)	−0.0004** (−2.5302)	−0.0005*** (−3.1246)	−0.0021*** (−2.7127)	−0.0007*** (−3.9559)	−0.0006*** (−3.5752)	−0.0007*** (−3.9559)	−0.0021*** (−2.7474)
*Cases*	−0.0032 (−1.0362)	0.0028 (0.9718)	−0.0032 (−1.0362)	0.0009*** (2.6839)				
*Deaths*					−0.0006 (−0.1773)	−0.0032 (−0.8949)	−0.0006 (−0.1773)	0.0005** (2.2233)
*Highly rated ESG firm*_*2019*_ * *Cases*	−0.0015 (−0.4833)	−0.0019 (−0.6458)	−0.0015 (−0.4833)	−0.0019*** (−3.3563)				
*Highly rated ESG firm*_*2019*_ * *Deaths*					0.0013 (0.4569)	0.0019 (0.6564)	0.0013 (0.4569)	−0.0011*** (−3.0074)
Intercept	Yes	Yes	Yes	Yes	Yes	Yes	Yes	Yes
Observations	72,798	72,798	72,798	72,786	72,798	72,798	72,798	72,786
*R*‐squared	0.296	0.083	0.313	0.084	0.296	0.083	0.313	0.084
Number of firms	375	375	375	375	375	375	375	375
Sector FEs	Yes	Yes	Yes	Yes	Yes	Yes	Yes	Yes
Day FEs	Yes	Yes	Yes	Yes	Yes	Yes	Yes	Yes

*Note*: This table shows the panel regression results for stock returns and volatility for the period January 2020 to December 2020. Panel A reports the results for non‐financial firms. Panel B reports the results for non‐financial firms by excluding oil‐ and energy‐related firms. Panel C reports the results for financial firms. The dependent variables are daily log‐returns (*Raw returns*), market‐adjusted returns (*Market‐adjusted returns*), excess returns (*Excess returns*), and the 5‐day rolling return volatility (*Volatility*), respectively. *Highly rated ESG firm*_*2019*_ is a dummy variable taking the value of one if the firm has a ESG rating higher than the median ESG score of the population of listed firms in the year before the COVID‐19 pandemic (2019). *Cases* variable is the daily log‐growth of confirmed coronavirus cases in the country *c* in the day *t*. *Deaths* is the daily log‐growth of deaths in the country *c* in the day *t*. Regressions include day and sector fixed effects because ESG ratings differ across sectors. Robust *t* statistics are reported in parentheses.

* *p* < 0.1.

** *p* < 0.05.

*** *p* < 0.01.

Panels A and B report the results for non‐financial firms. Panel C presents the results related to financial firms. However, we also report the results excluding observations referring to the energy‐ and oil‐related companies. This exercise is also coherent with the results of Kumar et al. (2022). On the one hand, this sector is more likely to pursue less environmentally friendly practices. On the other hand, Mazur et al. (2020) suggest that oil‐ and energy‐related firms suffered more than other firms from a sharp drop in oil prices^14^ at the beginning of the pandemic.

First, we observe that non‐financial firms with higher ESG scores have a better stock market performance than firms with lower ESG‐score when COVID 19‐related deaths and cases increase. The coefficient on *Highly rated ESG firm*_*2019*_ * *Cases* in Column 1 suggests that an increase by one standard deviation in the log‐growth of COVID‐19 cases increases *Raw Returns* by 0.06 percentage points at daily basis for non‐financial firms with higher ESG scores. Given an investment horizon of 1 year (260 trading days), we obtain that during the whole 2020 firms with higher ESG scores gain almost 91 percentage points (0.91%). Similar results are obtained when we consider COVID‐19 deaths (152 percentage points), when we exclude oil‐ and energy‐related firms, and when we rely on alternative specifications. The removal of oil‐ and energy‐related firms' observations increase the magnitude of our estimates.

Second, when we consider only financial firms, we do not find any evidence that financial firms with higher ESG scores outperform other firms in terms of stock price returns. However, we find that more sustainable financial firms have lower volatilities than other firms. For instance, the coefficient on *Highly rated ESG firm*_*2019*_ * *Cases* in Column 1 suggests that an increase by one standard deviation in the log‐growth of COVID‐19 cases would decrease *Volatility* by 0.02 percentage points at daily basis for firms with higher ESG scores (annually −32 percentage points).

Overall, our evidence suggests that non‐financial firms with a higher ESG scores benefit from better returns and lower risk than other firms. However, we do not find evidence that financial firms with higher ESG scores benefit from better returns. However, they show lower volatilities.

#### 4.2.3 Controlling for Fama French factors, momentum, and government policies

Thus far, we have correlated the firm's stock performance with the national COVID‐19 cases and deaths by allowing for systematic differences across sector types and firm heterogeneity. However, other daily firm‐specific economic conditions might drive our results. We address this issue in two ways. First, we include the Fama French factors and the momentum factor for European stocks.^15^ Second, we include daily proxies for public authorities' activities to backstop their national economies, such as *stay‐at‐home* measures and the economic support provisions to households and firms (Narayan et al., 2021; Ramelli & Wagner, 2020). The theoretical underpinning behind this test is that public authorities aim at avoiding generalized defaults in the economy (DeBandt & Hartmann, 2000), while investors might discount such an information in the asset prices (Aït‐Sahalia et al., 2012; Baig et al., 2021; Heyden & Heyden, 2021), Thus, we control for three daily measures related to how national governments have acted upon the economy and to what extent. We include the Containment and Health Index,^16^ Stringency Index,^17^ and the Economic Support Index^18^ (Hale et al., 2021).^19^ ^,^ ^20^ ^,^ ^21^

Table 7 presents the results. Our main results remain unaltered: highly rated ESG score firms have a better returns and lower volatilities. According to our more conservative estimates related to the coefficient on *Highly rated ESG firm*_*2019*_ * *Cases* in Column 1, it suggests that an increase by one standard deviation in the log‐growth of COVID‐19 cases would increase *Raw Returns* by 0.04 percentage points at daily basis for firms with higher ESG scores (annually, 59 percentage points). When moving to the results for the *Volatility*, the same coefficient suggests a daily decrease of 0.01 percentage points, that if converted over a horizon of 260 trading days (1 year), we obtain an annual decrease of 17 percentage points. We obtain comparable findings when we consider *Deaths*.

**TABLE 7:** Robustness tests: Allowing for Fama French factors, momentum, and government policies

Variables	Raw returns	Market‐adjusted returns	Excess returns	Volatility	Raw returns	Market‐adjusted returns	Excess returns	Volatility
	(1)	(2)	(3)	(4)	(5)	(6)	(7)	(8)
*Highly rated ESG firm*_*2019*_	−0.0003** (−2.3762)	−0.0002** (−2.1882)	−0.0003** (−2.2680)	−0.0041*** (−4.1045)	−0.0004*** (−3.1634)	−0.0003*** (−2.8710)	−0.0003*** (−2.6038)	−0.0041*** (−4.1109)
*Cases*	−0.0069*** (−5.1437)	0.0017 (1.2618)	−0.0209*** (−14.8272)	−0.0022*** (−8.5820)				
*Deaths*					−0.0079*** (−6.0965)	−0.0035*** (−2.6913)	−0.0177*** (−13.5440)	−0.0012*** (−6.3025)
*Highly rated ESG firm*_*2019*_ * *Cases*	0.0035* (1.7273)	0.0035* (1.7356)	0.0045** (2.2204)	−0.0010** (−2.2651)				
*Highly rated ESG firm*_*2019*_ * *Deaths*					0.0047** (2.5134)	0.0046** (2.4721)	0.0041** (2.2263)	−0.0009** (−2.5232)
*Rm‐Rf*	0.0105*** (94.6631)	0.0014*** (12.3247)	0.0104*** (93.9180)	0.0000*** (9.1785)	0.0105*** (95.7625)	0.0013*** (11.8160)	0.0104*** (94.8873)	0.0000*** (8.8933)
*SMB*	0.0089*** (30.4383)	0.0095*** (36.2145)	0.0090*** (31.2396)	0.0000*** (5.1402)	0.0088*** (30.5995)	0.0093*** (36.0275)	0.0088*** (31.0522)	0.0000*** (4.8968)
*HML*	0.0010*** (2.9176)	−0.0002 (−0.5294)	0.0015*** (4.5139)	0.0000*** (5.0103)	0.0007** (2.2824)	−0.0003 (−1.0146)	0.0010*** (3.0616)	0.0000 (0.1173)
*RMW*	0.0007 (1.5975)	−0.0003 (−0.6125)	0.0009** (2.0243)	0.0001*** (4.0188)	0.0007* (1.6885)	−0.0003 (−0.6973)	0.0010** (2.3675)	0.0001*** (5.2577)
*CMA*	−0.0003 (−0.7340)	−0.0004 (−1.2370)	−0.0010*** (−2.9940)	0.0000*** (3.8073)	−0.0003 (−0.9632)	−0.0006* (−1.7123)	−0.0011*** (−3.2278)	0.0000*** (4.6051)
*WML*	−0.0005*** (−2.9394)	0.0004** (2.2303)	−0.0004*** (−2.6848)	0.0000*** (6.7465)	−0.0006*** (−3.8666)	0.0002 (1.4529)	−0.0007*** (−4.4873)	0.0000*** (2.8107)
*Stringency Index*	0.0009 (0.9184)	−0.0025** (−2.4765)	−0.0081*** (−7.5914)	−0.0044*** (−5.1155)	0.0029*** (2.6025)	−0.0000 (−0.0393)	−0.0046*** (−3.8941)	−0.0044*** (−5.3355)
*Health Index*	0.0046*** (2.8277)	0.0065*** (4.0856)	0.0193*** (11.5637)	0.0086*** (5.9639)	0.0019 (1.1672)	0.0030* (1.8482)	0.0150*** (8.5549)	0.0087*** (6.2602)
*Economic index*	−0.0004* (−1.6577)	−0.0013*** (−5.0877)	0.0017*** (5.9208)	0.0017*** (15.0125)	−0.0006** (−2.4469)	−0.0018*** (−7.0169)	0.0016*** (5.5340)	0.0018*** (16.4840)
Intercept	Yes	Yes	Yes	Yes	Yes	Yes	Yes	Yes
Observations	227,923	227,923	227,923	227,864	227,923	227,923	227,923	227,864
*R*‐squared	0.242	0.026	0.256	0.019	0.242	0.026	0.257	0.019
Number of firms	1,204	1,204	1204	1,204	1,204	1,204	1,204	1,204

*Note*: This table shows the panel regression results for stock returns and volatility for the period January 2020 to December 2020. The dependent variables are daily log‐returns (*Raw returns*), market‐adjusted returns (*Market‐adjusted returns*), excess returns (*Excess returns*), and the 5‐day rolling return volatility (*Volatility*), respectively. *Highly rated ESG firm*_*2019*_ is a dummy variable taking the value of one if the firm has a ESG rating higher than the median ESG score of the population of listed firms in the year before the COVID‐19 pandemic (2019). *Cases* variable is the daily log‐growth of confirmed coronavirus cases in the country *c* in the day *t*. *Deaths* is the daily log‐growth of deaths in the country *c* in the day *t*. Regressions include Fama French factors (*Rm‐rf*, *SMB*, *HML*, *RMW*, and *CMA*) plus the momentum (*WML*) factor. We also include Stringency Index (*Stringency Index*), Containment and Health Index (*Health Index*), and Economic Support Index (*Economic Index*) from Oxford Covid‐19 Government Response Tracker (https://www.bsg.ox.ac.uk/research/research‐projects/covid‐19‐government‐response‐tracker). Robust *t* statistics are reported in parentheses.

* *p* < 0.1.

** *p* < 0.05.

*** *p* < 0.01.

The Fama French factors enter the regressions with coherent signs. Particularly, our results suggest that better (worse) economic conditions should lead to higher (lower) returns. While moving onto the government activities' proxies, we find that investors do not welcome stringency and economic measures. They attract negative stock market performance (Baig et al., 2021; Bannigidadmath et al., 2022). Conversely, containment and health measures attract positive returns in line with the perspective that investors positively evaluate investments in vaccines and healthcare systems (Rouatbi et al., 2021).

#### 4.2.4 Dynamic endogeneity

In this subsection, we are concerned about the potential dynamic endogeneity. Although COVID‐19 cases and deaths are plausibly exogenous to the corporate policies, there might exist a potential dynamic endogeneity (Chhaochharia & Laeven, 2009) in the stock price time‐series. For this reason, we perform a generalized method of moments (GMM) panel data technique to alleviate such concerns. First, we assume cases, deaths, and other regressors entering our econometric setup as predetermined values. Second, we lag our dependent variable by 2 days to obtain valid instruments (Chhaochharia & Laeven, 2009), and finally, we use the dependent variable's first two lags as explanatory variables to run a dynamic model (Arellano & Bond, 1991).

We report the results in Table 8. Once again, we find that the stock market performance of more sustainable firms is better than others when the COVID‐19 cases and deaths increase in the national economies. For the sake of brevity, we report only the estimates related to the COVID‐19 cases.^22^ Similar findings are obtained when we opt for sample splits (financial vs. non‐financial firms) and drop oil‐related firm observations from our sample.

**TABLE 8:** Robustness tests: GMM models

	Full sample				Panel A: Non‐financial firms			
Variables	Raw returns	Market‐adjusted returns	Excess returns	Volatility	Raw returns	Market‐adjusted returns	Excess returns	Volatility
	(1)	(2)	(3)	(4)	(5)	(6)	(7)	(8)
*Raw returns*_*t‐1*_	0.1158*** (11.6709)				0.1155*** (9.2512)			
*Raw returns*_*t‐2*_	0.0555*** (11.4776)				0.0481*** (7.8756)			
*Market‐adjusted returns*_*t‐1*_		0.0430** (2.4665)				0.0339** (2.4461)		
*Market‐adjusted returns*_*t‐2*_		0.0133** (2.4900)				0.0155** (2.2819)		
*Excess returns*_*t‐1*_			0.1453*** (15.5156)				0.1457*** (12.3767)	
*Excess returns*_*t‐2*_			0.0614*** (12.4907)				0.0546*** (8.7901)	
*Volatility*_*t‐1*_				1.1703*** (25.5060)				1.1895*** (21.2818)
*Volatility*_*t‐2*_				0.1727*** (3.7585)				0.1912*** (3.4182)
*Highly rated ESG firm*_*2019*_	−0.0004*** (−3.0059)	−0.0003** (−2.2438)	−0.0005*** (−3.2943)	−0.0000 (−0.1620)	−0.0004* (−1.9522)	−0.0002 (−1.3214)	−0.0004** (−2.1570)	−0.0000 (−0.0341)
*Cases*	−0.0479*** (−21.8389)	−0.0090*** (−4.7844)	−0.0608*** (−24.9980)	0.0003*** (10.6900)	−0.0496*** (−16.8005)	−0.0098*** (−4.0408)	−0.0612*** (−18.9519)	0.0003*** (8.3526)
*Highly rated ESG firm*_*2019*_ * *Cases*	0.0076** (2.4016)	0.0040 (1.5335)	0.0091*** (2.6385)	−0.0001*** (−4.8377)	0.0100** (2.4129)	0.0062* (1.8459)	0.0106** (2.3690)	−0.0002*** (−4.2186)
Intercept	Yes	Yes	Yes	Yes	Yes	Yes	Yes	Yes
Observations	226,884	225,846	226,884	226,808	154,422	153,720	154,422	154,361
Number of firms	1,204	1,204	1,204	1,200	829	829	829	826
Hansen test (*p*‐value)	0.913	0.917	0.919	0.855	1.000	1.000	1.000	1.000
AR (1) *p* value	0.000	0.000	0.000	0.000	0.000	0.000	0.000	0.000
AR (2) *p* value	0.000	0.000	0.000	0.005	0.000	0.000	0.000	0.012
Sample	Full sample	Full sample	Full sample	Full sample	Non‐financials	Non‐financials	Non‐financials	Non‐financials

	Panel B: Non‐financial firms with the exclusion of oil and energy‐related firms				Panel C: Financial firms			
Variables	Raw returns	Market‐adjusted returns	Excess returns	Volatility	Raw returns	Market‐adjusted returns	Excess returns	Volatility
	(9)	(10)	(11)	(12)	(13)	(14)	(15)	(16)
*Raw returns*_*t‐1*_	0.1226*** (10.4344)				0.0940*** (6.7703)			
*Raw returns*_*t‐2*_	0.0482*** (7.5324)				0.0709*** (10.5246)			
*Market‐adjusted returns*_*t‐1*_		0.0561*** (2.7948)				0.0324* (1.8171)		
*Market‐adjusted returns*_*t‐2*_		0.0157** (2.1903)				0.0115* (1.8273)		
*Excess returns*_*t‐1*_			0.1509*** (13.3941)				0.1217*** (9.1044)	
*Excess returns*_*t‐2*_			0.0550*** (8.3970)				0.0760*** (11.0571)	
*Volatility*_*t‐1*_				1.1879*** (20.7752)				1.1341*** (24.1916)
*Volatility*_*t‐2*_				0.1897*** (3.3138)				0.1391*** (2.9991)
*Highly rated ESG firm*_*2019*_	−0.0003* (−1.7836)	−0.0002 (−1.2775)	−0.0004** (−1.9836)	0.0000 (0.4175)	−0.0005** (−2.2834)	−0.0004* (−1.8801)	−0.0006** (−2.3936)	−0.0000 (−0.1908)
*Cases*	−0.0495*** (−17.8923)	−0.0097*** (−4.0258)	−0.0610*** (−20.2185)	0.0003*** (8.4430)	−0.0444*** (−15.5276)	−0.0069** (−2.5757)	−0.0592*** (−17.4532)	0.0003*** (7.8175)
*Highly rated ESG firm*_*2019*_ * *Cases*	0.0086** (2.0441)	0.0051 (1.5133)	0.0094** (2.0882)	−0.0001*** (−4.0584)	0.0009 (0.1973)	−0.0017 (−0.4281)	0.0033 (0.6161)	−0.0001* (−1.8784)
Intercept	Yes	Yes	Yes	Yes	Yes	Yes	Yes	Yes
Observations	148,241	147,561	148,241	148,180	72,462	72,126	72,462	72,447
Number of firms	790	790	790	787	375	375	375	374
Hansen test (*p* value)	1.000	1.000	1.000	1.000	1.000	1.000	1.000	1.000
AR (1) *p* value	0.000	0.000	0.000	0.000	0.000	0.000	0.000	0.000
AR (2) *p* value	0.000	0.000	0.000	0.009	0.000	0.000	0.000	0.051
Sample	No Oil and Gas	No Oil and Gas	No Oil and Gas	No Oil and Gas	Financials	Financials	Financials	Financials

*Note*: This table reports the results for the two‐step GMM estimates for stock returns and volatility for the period January 2020 to December 2020. The dependent variables are daily log‐returns (*Raw returns*), market‐adjusted returns (*Market‐adjusted returns*), excess returns (*Excess returns*), and the 5‐day rolling return volatility (*Volatility*) respectively. We also report the dynamics of the dependent variables (two lags). *Highly rated ESG firm*_*2019*_ is a dummy variable taking the value of one if the firm has a ESG rating higher than the median ESG score of the population of listed firms in the year before the COVID‐19 pandemic (2019). *Cases* variable is the daily log‐growth of confirmed coronavirus cases in the country *c* in the day *t*. Columns (1) to (4) report the results for the full sample. Columns (5) to (8) report the results for non‐financial firms (panel A). Columns (9) to (12) report the results for non‐financial firms by excluding oil‐ and energy‐related firms (panel B). Columns (13) to (16) report the results for financial firms (panel C). Robust *t* statistics are reported in parentheses.

* *p* < 0.1.

** *p* < 0.05.

*** *p* < 0.01.

#### 4.2.5 Market liquidity of highly rated ESG score firms

This subsection illustrates how more sustainable firms' stock liquidity correlates with the pandemic spread. Stock market liquidity represents the efficiency degree that investors may easily convert such stocks into cash without incurring losses in the stock market value. This feature matters for investors, especially during the adverse states of the economy, where the increased volatility and uncertainty deteriorate the overall liquidity of financial markets (Baig et al., 2021).

We focus on the bid‐ask spread because as well as being suitable for many applications ranging from corporate finance to asset pricing and macroeconomics studies (Hasbrouck, 2009; Fang et al., 2009; Korajczyk & Sadka, 2008; Næs et al., 2011), it is also appropriate to detect potential frictions between the demand and supply sides for a given specific stock. In addition, this measure appears to be easier to interpret since more liquid stocks are more likely to show lower bid‐ask spreads. We follow a similar specification in line with our evidence presented in Sections 4.1 and 4.2. Table 9 reports the results.

**TABLE 9:** COVID cases and deaths growth and stock market liquidity of highly rated ESG stocks

Panel A						
Variables	Bid ask spread	Bid ask spread	Bid ask spread	Bid ask spread	Bid ask spread	Bid ask spread
	(1)	(2)	(3)	(4)	(5)	(6)
*Highly rated ESG firm*_*2019*_	0.0899 (1.2400)	0.0919 (1.2668)	0.0084 (0.0832)	0.01 (0.0993)	0.0091 (0.0863)	0.0107 (0.1012)
*Cases*	0.0222 (0.9617)		0.0144 (0.4781)		0.0147 (0.4651)	
*Deaths*		0.0754*** (4.6148)		0.0674*** (3.2554)		0.0718*** (3.2993)
*Highly rated ESG firm*_*2019*_ * *Cases*	−0.0729** (−2.4464)		−0.0821** (−2.0459)		−0.0883** (−2.0819)	
*Highly rated ESG firm*_*2019*_ * *Deaths*		−0.0931*** (−3.9335)		−0.0936*** (−3.0159)		−0.0987*** (−3.0266)
Intercept	Yes	Yes	Yes	Yes	Yes	Yes
Observations	217,802	217,802	148,345	148,345	142,368	142,368
*R*‐squared	0.016	0.016	0.012	0.012	0.009	0.009
Number of firms	1,194	1,194	824	824	785	785
Sector FEs	Yes	Yes	Yes	Yes	Yes	Yes
Firms FEs	No	No	No	No	No	No
Day FEs	Yes	Yes	Yes	Yes	Yes	Yes
Sample	Full sample	Full sample	Non‐financials	Non‐financials	No Oil and Gas	No Oil and Gas

Panel B						
Variables	Bid ask spread	Bid ask spread	Bid ask spread	Bid ask spread	Bid ask spread	Bid ask spread
	(7)	(8)	(9)	(10)	(11)	(12)
*Highly rated ESG firm*_*2019*_	0.2740*** (4.3846)	0.2766*** (4.3956)			0.0998 (1.3944)	0.1019 (1.4229)
*Cases*	0.0360 (1.0662)		0.0219 (0.9515)		0.0209 (0.9408)	
*Deaths*		0.0948*** (3.6233)		0.0756*** (4.6239)		0.0479*** (2.9668)
*Highly rated ESG firm*_*2019*_ * *Cases*	−0.0561 (−1.5395)		−0.0732** (−2.4574)		−0.0691** (−2.4375)	
*Highly rated ESG firm*_*2019*_ * *Deaths*		−0.0933*** (−2.9272)		−0.0933*** (−3.9439)		−0.0927*** (−4.0013)
*Rm‐rf*					0.0033*** (4.9404)	0.0039*** (5.5518)
*SMB*					0.0038** (2.3689)	0.0055*** (3.2667)
*HML*					0.0059*** (2.9975)	0.0072*** (3.6407)
*RMW*					−0.0006 (−0.1283)	−0.0003 (−0.0617)
*CMA*					0.0013 (0.5763)	0.0027 (1.2214)
*WML*					0.0065*** (6.4038)	0.0076*** (6.7630)
*Stringency Index*					−0.0566 (−0.8698)	−0.0896 (−1.3827)
*Health Index*					0.0421 (0.4538)	0.0923 (0.9980)
*Economic Index*					−0.0836*** (−5.2002)	−0.0719*** (−4.7137)
Intercept	Yes	Yes	Yes	Yes	Yes	Yes
Observations	69,457	69,457	217,802	217,802	217,802	217,802
*R*‐squared	0.052	0.052	0.005	0.005	0.002	0.002
Number of firms	370	370	1,194	1194	1194	1,194
Sector FEs	Yes	Yes	No	No	No	No
Firms FEs	No	No	Yes	Yes	No	No
Day FEs	Yes	Yes	Yes	Yes	No	No
Sample	Financials	Financials	Full sample	Full sample	FF and policy indicators	FF and policy indicators

*Note*: This table shows the panel regression results for the stock liquidity for the period January 2020 to December 2020. The dependent variable is the bid‐ask spread (*Bid‐ask spread*). *Highly rated ESG firm*_*2019*_ is a dummy variable taking the value of one if the firm has a ESG rating higher than the median ESG score of the population of listed firms in the year before the COVID‐19 pandemic (2019). *Cases* variable is the daily log‐growth of confirmed coronavirus cases in the country *c* in the day *t*. *Deaths* is the daily log‐growth of deaths in the country *c* in the day *t*. Columns (1) and (2) report the results for the full sample. Columns (3) and (4) report the results for non‐financial firms (panel A). Columns (5) and (6) report the results for non‐financial firms by excluding oil‐ and energy‐related firms (panel B). Columns (7) and (8) report the results for financial firms (panel C). Regressions include day, sector and (only for columns (9) and (10)) firms fixed effects because ESG ratings differ across sectors. Regressions in columns (11) and (12) include also Fama French factors (*Rm‐rf*, *SMB*, *HML*, *RMW*, and *CMA*), momentum factor (*WML*) and Stringency Index (*Stringency Index*), Containment and Health Index (*Health Index*), and Economic Support Index (*Economic Index*) from Oxford Covid‐19 Government Response Tracker (https://www.bsg.ox.ac.uk/research/research‐projects/covid‐19‐government‐response‐tracker). Robust *t* statistics are reported in parentheses.

* *p* < 0.1.

** *p* < 0.05.

*** *p* < 0.01.

Except for Column 7, the results consistently show that when the number of COVID‐19 cases and deaths increases in the economy firms with higher ESG scores have lower bid‐ask spread suggesting that these stocks have higher market liquidity.

#### 4.2.6 Determinants of resilience of more sustainable firms

Thus far, we have explored more sustainable firms' stock‐market performance and stock liquidity. In this section, we answer to another correlated question: Why do some more sustainable firms perform better than other firms during the pandemic?

Hence, we explore the firm‐specific characteristics in the pre‐COVID‐19 period and focus on two potential and plausible channels (not mutually exclusive) explaining why some highly rated ESG score firms might perform better than other firms. The first channel we refer to is the one we define as the *liquidity channel*. This channel finds its foundation in the paper of Ding et al. (2021) and Albuquerque et al. (2020). The authors consistently demonstrate that the better market performance of some firms during the pandemic might depend on the ex‐ante liquidity conditions. In this way, these firms should be more likely to absorb economic shocks. In our setup, we check whether firms with higher ESG evaluations, more cash‐holdings, and liquid assets in the pre‐COVID period (2019) have better performance during the pandemic.

Second, we recognize the existence of a *performance channel*. The literature on the performance channel posit two alternative explanations. On the one hand, Beltratti and Stulz (2012) suggest that the better stock market performance in the financial crisis might be due to a reversal in the pre‐crisis performance. Thus, in line with this argument, we might also speculate that firms with higher ESG scores and higher performance might be bad performers in the pre‐COVID period. On the other hand, the alternative view advocates that the firms with higher ESG scores perform better during the pandemic because they were already more profitable than other firms in the pre‐pandemic period. The consideration of such an argument is also important because it might help to exclude potential self‐selection biases.^23^

For this further analysis, we estimate the firm stock market performance during the whole year of 2020 as a function of the pre‐pandemic firm characteristics (2019). We first rely on the one‐year buy‐and‐hold stock returns (Erkens et al., 2012; Griffin & Lemmon, 2002) related to 2020. This measure depicts the expected return of an investor if she/he bought the stock on the first day of January and held it until the last day of December 2020.^24^ Then, we regress this variable on the firm‐specific characteristics including firm cash‐holdings and short‐term investments, profitability, size, fixed assets, and leverage as well as the firm performance sustainability. We also introduce two interactive terms, obtained as the product between firm profitability and firm performance sustainability (*Highly rated ESG firm*_*2019*_ dummy), and the interaction between firm liquidity conditions and firm performance sustainability. Since we run a cross‐sectional analysis, we include country and sector dummies to account for the within‐sector and within‐country variation in the corporate traits. Our estimation procedure is as follows:

(7) $${\mathrm{BHR}}_{2020}=b_{0}+b_{1}\;\text{Highly}-\text{rated}\;\mathrm{ESG}\;{\text{firm}}_{2019}+b_{2}\;{\text{Size}}_{2019}+{b_{3}}^{*}\;{\text{Debt Ratio}}_{2019}+b_{4}\;{\mathrm{ROA}}_{2019}+b_{5}\;{\text{Cash Ratio}}_{2019}+b_{6}\;{\text{Fixed Ratio}}_{2019}+b_{7}\;{{\text{Cash Ratio}}_{2019}}^{*}\;\text{Highly}-\text{rated}\;\mathrm{ESG}\;{\text{firm}}_{2019}+b_{8}\;{{\mathrm{ROA}}_{2019}}^{*}\;\text{Highly}-\text{rated}\;\mathrm{ESG}\;{\text{firm}}_{2019}+e_{i,t}$$

where *BHR*_*2020*_ stands for buy‐and‐hold stock returns, while *Cash Ratio*_*2019*_ * *Highly rated ESG firm*_*2019*_ and *ROA*_*2019*_ * *Highly rated ESG firm*_*2019*_ are the variables of our interest. *ROA*_*2019*_ stands for the return on assets, while *Cash Ratio*_*2019*_ is defined as the total amount of cash and liquid assets divided by total assets (Ding et al., 2021). We also include a standard set of control variables, such as *Size*_*2019*_ *(*the log of the firm's total assets), *Fixed Ratio*_*2019*_ (the ratio between total fixed assets and total assets), and *Debt Ratio*_*2019*_ (the ratio between total debt and total assets) to account for the firm's structure of its assets, leverage, and performance in the pre‐COVID 19 period.^25^ Table 10 reports the results.

**TABLE 10:** Buy‐and‐hold stock return analysis

	BHR_2020_	BHR_2020_	BHR_2020_	BHR_2020_	BHR_2020_	BHR_2020_
Variables	(1)	(2)	(3)	(4)	(5)	(6)
*Highly rated ESG firm*_*2019*_	−0.0345 (−1.1761)	−0.1720*** (−3.8536)	0.0183 (0.4562)	0.0006 (0.0203)	−0.0683 (−1.6145)	0.0283 (0.6984)
*Size*_*2019*_	−0.0110* (−1.7093)	−0.0057 (−0.5553)	−0.0108 (−1.2907)	−0.0228*** (−3.9566)	−0.0294*** (−3.3883)	−0.0101 (−1.1831)
*Debt Ratio*_*2019*_	−0.0960* (−1.8184)	−0.2957*** (−3.5059)	−0.0409 (−0.6038)	−0.1559*** (−2.9857)	−0.3631*** (−4.6204)	−0.0917 (−1.3251)
*ROA*_*2019*_	0.8796*** (8.5472)	0.0468 (0.4023)	0.8273*** (4.4305)	0.6916*** (6.8334)	0.0686 (0.6392)	0.7151*** (3.7920)
*Cash Ratio*_*2019*_	−0.2217** (−2.3914)	0.1226 (0.9261)	−0.2834** (−2.1775)	−0.1321 (−1.4329)	0.1212 (0.9738)	−0.2657** (−2.0169)
*Fixed Ratio*_*2019*_	−0.0230 (−0.4841)	0.1695** (2.0855)	−0.0849 (−1.4460)	−0.0308 (−0.8537)	0.0068 (0.1399)	−0.0587 (−1.0922)
*Cash Ratio*_*2019*_ * *Highly rated ESG firm*_*2019*_	0.7179*** (4.1998)	0.4658** (2.0406)	0.5634** (2.2782)	0.6721*** (4.0302)	0.4484** (2.1205)	0.5409** (2.1921)
*ROA*_*2019*_ * *Highly rated ESG firm*_*2019*_	−0.2156 (−0.9718)	0.3219 (1.0887)	−0.0592 (−0.1788)	−0.3219 (−1.4773)	0.0576 (0.2110)	−0.3281 (−0.9867)
Intercept	Yes	Yes	Yes	Yes	Yes	Yes
Observations	1,203	374	829	1,203	374	829
R‐squared	0.193	0.232	0.155	0.236	0.372	0.198
Fixed Effect	Sector	Sector	Sector	Country	Country	Country
Sample	All sample	Financial	Non‐financial	All sample	Financial	Non‐financial

*Note*: This table reports the results for the relation between firm performance, ESG score, and firm‐specific characteristics. The dependent variable is the buy‐and‐hold stock returns (*BHR*) during the whole COVID‐19 crisis (Erkens et al., 2012). *Highly rated ESG firm*_*2019*_ is a dummy variable taking the value of one if the firm has a ESG rating higher than the median ESG score of the population of listed firms in the year before the COVID‐19 pandemic (2019). All the controls are referred to the period before the COVID‐19 crisis (accounting year: 2019). *Size*_*2019*_ is the log of the firms total assets. *Debt Ratio*_*2019*_ is the ratio between total debt and total assets. *ROA*_*2019*_ is the return to assets. *Cash Ratio*_*2019*_ is the total amount of Cash and liquid assets divided by total assets. *Fixed Ratio*_*2019*_ is the ratio between total fixed assets and total assets. Intercept included but not reported. Specifications include sector‐fixed effects and country‐fixed effects. Robust *t* statistics are reported in parentheses.

* *p* < 0.1.

** *p* < 0.05.

*** *p* < 0.01.

Our finding supports the view that the *ex‐ante* liquidity conditions matter to explain firm stock market performance. In fact, the coefficient on *Cash Ratio*_*2019*_ * *Highly rated ESG firm*_*2019*_ is positively and statistically significant across all specifications (5% or better), suggesting that firms with higher ESG scores and higher ex‐ante liquidity conditions perform better than other firms. This evidence is consistent with the idea that higher cash holdings permit to absorb the pandemic crash. However, the ex‐ante liquidity conditions matter more for financial firms.

**FIGURE 1:**
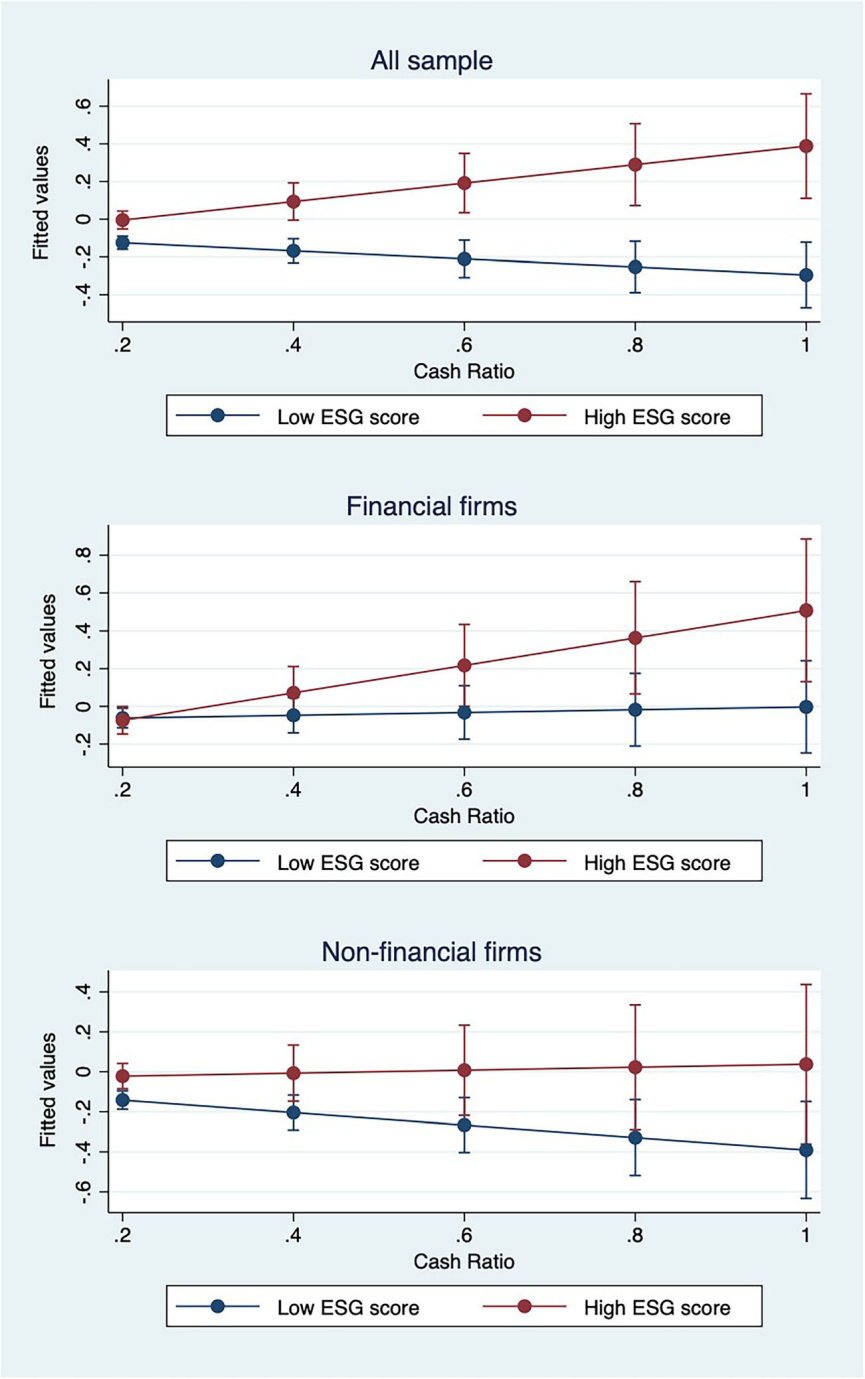
Marginal effects of Cash Ratio on the firm buy‐and‐hold stock returns. This figure shows the predicted marginal effect of Cash Ratio on the buy‐and‐hold stock returns for specified thresholds (0.2, 0.4, 0.6, 0.8, and 1—extreme case in which the amount of cash‐holdings and liquid assets equals the amount of the total assets) of *Cash Ratio*

In terms of economic magnitude of our results, if we consider more conservative estimates (Column 4) referring to the whole sample, we obtain that one standard increase in the firm pre‐COVID period of cash holdings is associated with an increase in the buy‐and‐hold stock returns by approximately 8.14 percentage points. We also provide some evidence on the different sensitivity of the buy‐and‐hold stock returns (predicted marginal effect) to several thresholds^26^ of *Cash Ratio*. Figure 1 highlights the results indicating that ex‐ante liquidity conditions are more prominent for financial firms than non‐financial firms to absorb economic shock, such as the COVID‐19 pandemic.

Next, our results exclude the existence of a *performance channel*. Indeed, the coefficient on the interactive term—*ROA*_*2019*_ * *Highly‐rated ESG firm*_*2019*_—enters all regressions with a non‐significant coefficient. This is also important because it excludes that better performing firms with higher ESG scores in the pre‐COVID are more likely to perform better during 2020.

## 5 CONCLUSIONS

While recent academic studies have investigated the implications of COVID‐19 pandemic shock on the financial markets (among others, Albuquerque et al., 2020; Erdem, 2020; Ramelli & Wagner, 2020; Zhang et al., 2020), there is currently no comprehensive evidence about the linkage between the COVID‐19 pandemic shock, firm sustainability, and stock market performance.

Exploiting an extensive data set of 1,204 (financial and non‐financial) firms from 15 European countries, we show that firms with higher ESG scores perform better than low‐rated ESG firms when public authorities announce their national number of confirmed cases and deaths due to the COVID‐19. Then, after a host of robustness checks based on different specifications accounting for potential endogeneity, our results hold. Second, we also highlight the mechanism through which more sustainable firms fare better than other firms during the pandemic. Our findings exclude the existence of a performance channel, namely the post‐COVID performance of more sustainable firm is function of the pre‐COVID profitability. However, they underline that firms with higher ESG scores perform better than other firms if they retain higher cash holdings and liquid assets necessary to absorb the pandemic externalities.

On the one hand, our outcomes first contribute to the literature on the performance of high ESG score securities by highlighting their usual risk–return characteristics and stock market liquidity. On the other hand, we also contribute to the literature related to the channels through which more sustainable firms may build their resilience to unexpected shocks.

Hence, our results prescribe important implications for investors and firms. First, according to our estimates, investors consider firm sustainability as a valuable aspect since these firms show a better stock market performance during the COVID‐19 pandemic. In addition, they also benefit from a higher stock liquidity, which is another important attribute for securities' evaluation because the potential investment illiquidity may trigger and exacerbate the losses in investors' portfolios. This evidence is coherent with the need to analyze other aspects of the asset prices rather than the typical risk–return trade‐off in the investors' evaluation (Cunha et al., 2021).

Second, the orientation toward sustainability is a critical factor for firms to improve financial performance and generate shareholder value (Albuquerque et al., 2020), and more in general, all stakeholders' wealth (Porter & Kramer, 2006). On the one hand, by addressing investors' preferences toward sustainability, firms are likely to attract funds for their investments and support their growth processes. On the other hand, the integration of ESG factors and related risks in the business strategy and models might help them to build their resilience and increase their survival odds during adverse states of the economy. These insights might become increasingly relevant in case of similar and unexpected shocks. In this context, firms should accelerate their transition to more sustainable business models.

Nevertheless, our evidence suggests that firm sustainability on a stand‐alone basis does not ensure resilience and competitive advantage if not combined with sound financial fundamentals, such as a flexible financial structure. This aspect is also coherent with Broadstock et al. (2019) and points to discourage potential green‐washing practices (Arvidsson et al., 2022).

However, we recognize that there might be other channels through which firms may build their resilience, and this facet remains an open question for scholars. At the same time, we also acknowledge that the firm's liquidity is not the *panacea* for all the economic scenarios (Atif et al., 2022). During less adverse states of the economy, the retainment of excessive cash holdings and more liquid assets might increase the firm opportunity costs eroding profitability and growth opportunities. These considerations raise other relevant questions for scholars to explore.

Overall, our evidence corroborates current cross‐sectoral public policies to promote sustainable development concerning the energy, environment, economy, and development cooperation aimed at ensuring the resilience of the entire system.

## CONFLICT OF INTEREST

All the authors declare no conflict of interest.

## Supporting information


**Appendix S1.A** Main differences in the construction of ESG scores
**Appendix S1.B** Descriptive statistics for the cross‐sectional analysis for the buy‐and‐hold stock returns

## ENDNOTES

^1^
According to the Global Sustainable Investment Review (2020), the rise of ESG‐oriented investments topped 35 trillion dollars in 2020.

^2^

https://am.jpmorgan.com/sg/en/asset-management/per/insights/market-insights/on-the-minds-of-investors/covid-19-esg-matters/.

^3^
Scholars underline several difference between the United States and Europe, such as such as opaqueness and transparency of the financial markets (Gutiérrez & Philippon, 2018), degree of social inclusion in public services, and different degree of labor market stickiness (available at: https://www.clevelandfed.org/en/newsroom-and-events/publications/economic-commentary/economic-commentary-archives/2011-economic-commentaries/ec-201111-labor-market-rigidity-unemployment-and-the-great-recession.aspx).

^4^
One example is the Italian government that was the first national authority to impose stay‐at‐home measures (such as generalized lockdowns and quarantine measures) and restrict the movements of its habitants except for healthcare services and work commuting. Additionally, on March 19, 2020, the European Commission adopted a Temporary Framework for State aid measures that allows member states more flexibility to support the economy during the pandemic. On July 21, 2020, the European Council finally reached an agreement on The Next Generation EU (formerly denominated Recovery Plan), the largest stimulus package ever financed in Europe. The value of this plan is around 750 billion euros and is designed to boost the economic recovery through 360 billion euros in loans and 390 billion euros in grants (https://ec.europa.eu/info/strategy/recovery-plan-europe_en).

^5^

https://ec.europa.eu/info/strategy/priorities-2019-2024.

^6^
The stakeholder theory suggests that the better corporate financial performance may be ascribed to a higher firm's ability to manage a large array of stakeholders directly and indirectly linked to the firm actions, such as employees, civil society, customers, investors, and providers (Freeman & Reed, 1983).

^7^
The firm ability to achieve financial success depends on internal resources (for instance, employees and intangible assets).

^8^
Based on the assumption that there is a social contract between firms and the society, firms have some incentives to divulge their social responsibilities and commitment to their set of stakeholders to achieve a competitive advantage.

^9^
Some evidence supporting our hypothesis comes from the mutual fund literature. During periods of economic downturns, highly rated ESG mutual funds outperform than low rated ones (Hawn et al., 2018; Ruf et al., 2019).

^10^
We summarize the differences in the construction of ESG scores between the three leading providers in Appendix S1.A.

^11^
The inclusion of day fixed effects in our estimation procedure makes the estimates for *Raw Returns* and *Excess returns* equal. This circumstance is not verified when we include Fama and French factors rather than day fixed effects.

^12^
However, the coefficient becomes statistically significant at 5% or better for all the measures of stock market performance (*Raw returns*, *Market‐adjusted returns*, and *Excess returns*).

^13^
In our econometric setup, we rely on the following definition of corporate culture in line with Liao (2018), where the author refers to the corporate culture as “… the specific values and basic beliefs rooted in a firm that guides all the activities and behavior of that firm affecting innovation” (p. 1369) and performance.

^14^

https://www.forbes.com/sites/davidblackmon/2020/12/17/2020-in-review-one-of-the-oil-and-gas-industrys-worst-years-approaches-a-merciful-end/.

^15^
We consider the daily excess return on the market (*Rm‐rf*). We include the Small Minus Big factor (*SMB*), which is defined as the difference between the average return on small‐capitalization portfolios and the average return on large‐capitalization portfolios. Then, we consider High Minus Low factor (HML), calculated as the average return on portfolios of high book‐to‐market stocks minus the average return on portfolios of low book‐to‐market stocks. We also add the Conservative Minus Aggressive factor (CMA), namely the difference between the average returns on two conservative investments portfolios and the average return on the two aggressive investment portfolios and the Robust Minus Weak factor (RMW) defined as the average return on two robust operating profitability portfolios minus to which the average return of two weak operating profitability portfolios is subtracted. Finally, we also control for the momentum factor (WML) obtained as the difference between the equal‐weighted average return for two winner portfolios for the European region and the equal‐weighted average returns on two loser portfolios. The rationale for the inclusion of these variables is that better (worse) economic conditions should lead to higher (lower) returns (Carhart, 1997; Fama & French, 2015).

^16^
Containment and Health Index is a proxy for government activities in contact tracing, investments in the healthcare system and vaccines.

^17^
Stringency Index refers to how strong national governments impose lock‐down measures aimed at restricting people mobility within the national borders.

^18^
Economic Support Index considers all public policies aimed at supporting the households and corporates income.

^19^
For the construction of the indicators, visit the webpage: https://github.com/OxCGRT/covid-policy-tracker/blob/master/documentation/index_methodology.md.

^20^
For the sake of brevity, we rename these measures as *Stringency Index*, *Health Index*, and *Economic Index*.

^21^
All these variables are ordinal values ranging from 0 to 100, and the related distributions are positively skewed since their values become stronger during 2020.

^22^
The results related to COVID‐19 deaths are available upon request.

^23^
To exclude this argument, we stipulate that the coefficient on the variable of interest enters our regressions with a non‐significant sign.

^24^
If firms delist whenever earlier during the accounting year 2020.

^25^
We provide the summary statistics in Appendix S1.B.

^26^
We use the following threshold: 0.2, 0.4, 0.6, 0.8, and 1.00.
