# Supplementary material for: COVID‐19, ESG investing, and the resilience of more sustainable stocks: Evidence from European firms
Source: Bus Strategy Environ. 2022 Jun 8:10.1002/bse.3163. Online ahead of print. doi: 10.1002/bse.3163 (PMC9348247; doi:10.1002/bse.3163)
Supplement: Supplementary file 1 — Appendix S1.A Main differences in the construction of ESG scores Appendix S1.B Descriptive statistics for the cross‐sectional analysis for the buy‐and‐hold stock returns [file BSE-9999-0-s001.docx]

**Supplementary appendix**

**Not for publication**

**A.1. Main differences in the construction of ESG scores**

This table reports the basic constituent indicators used to construct ESG ratings among the main providers. Source: Dorfleitner et al. (2015), Berg et al. (2020), Widyawati (2020) and Cornell (2021).

|  | **Asset4** | **MSCI** | **KLD** |  |
| --- | --- | --- | --- | --- |
|  | |  |  |  |
| Access to basic services | | Yes | Yes | Yes |
| Access to healthcare system | | Yes | Yes | Yes |
| Accuracy of the audit system | | Yes | No | No |
| Animal welfare | | Yes | No | No |
| Anti-trust issues | | Yes | Yes | Yes |
| Board heterogeneity | | Yes | No | Yes |
| Board mechanisms | | Yes | Yes | No |
| Business Ethics | | Yes | Yes | Yes |
| Child workforce | | Yes | No | No |
| Chemical spills | | Yes | No | Yes |
| Clinical experimentations | | Yes | No | No |
| Control and reporting of GHG emissions | | Yes | Yes | Yes |
| Corruption events | | Yes | Yes | Yes |
| Customer relationship management (*CRM*) | | Yes | No | Yes |
| Ensuring biodiversity in productive processes | | Yes | Yes | Yes |
| Environmental efficiency | | Yes | Yes | No |
| Financial and social inclusion | | No | Yes | Yes |
| Green buildings | | Yes | No | Yes |
| Green range of products | | Yes | Yes | Yes |
| HIV policies | | Yes | No | No |
| Job actions | | Yes | No | No |
| Management of physical climate risks | | Yes | No | Yes |
| Ozone depletion | | Yes | No | No |
| Philanthropical services for the community | | Yes | No | Yes |
| Privacy and IT | | No | Yes | Yes |
| Processes of circular economy | | No | Yes | No |
| Product packaging | | No | Yes | Yes |
| Protection of human rights | | Yes | No | Yes |
| Safety of the products | | Yes | Yes | Yes |
| Sustainable finance | | Yes | Yes | Yes |
| Turnover in the workforce | | Yes | No | No |
| Water quality management | | Yes | Yes | Yes |
|  | |  |  |  |

**A.2. Descriptive statistics for the cross-sectional analysis for the buy-and-hold stock returns**

The table shows the descriptive statistics for the cross-sectional analysis based on the buy and hold stock returns. As before, for each variable, we show the following statistics: number of observations (Obs.), mean (“Average”), standard deviation (“Std. dev.”), minimum value (“Min.”) and maximum value (“Max.”). The buy-and-hold stock returns (*BHR*) during the whole COVID-19 crisis is calculated in line with Erkens et al. (2012). *Size_2019_* is the log of the firm’s total assets. *Debt Ratio_2019_* is the ratio between total debt and total assets. *ROA_2019_* is the return to assets. *Cash Ratio_2019_* is the total amount of Cash and liquid assets divided by total assets. *Fixed Ratio_2019_* is the ratio between total fixed assets and total assets.

| *Cross-sectional analysis in the robustness checks* | | | | | |
| --- | --- | --- | --- | --- | --- |
| ***Variable(s): All sample*** |  |  |  |  |  |
|  | *Obs.* | *Average* | *Std. dev.* | *Min.* | *Max.* |
| *BHR_2020_* | 1,204 | -0.0502 | 0.3940 | -0.9150 | 1.5146 |
| *Size_2019_* | 1,203 | 15.1983 | 1.9040 | 11.4287 | 20.5965 |
| *Debt Ratio_2019_* | 1,203 | 0.2685 | 0.1722 | 0.0092 | 0.7291 |
| *ROA_2019_* | 1,204 | 0.0627 | 0.0644 | -0.0776 | 0.3419 |
| *Cash Ratio_2019_* | 1,203 | 0.1108 | 0.1211 | 0.0000 | 0.9893 |
| *Fixed Ratio_2019_* | 1,204 | 0.2670 | 0.2585 | 0.0000 | 0.9923 |
|  |  |  |  |  |  |
